# Tissue and cellular localization of condensed tannins in poplar roots and potential association with nitrogen uptake

**DOI:** 10.3389/fpls.2024.1388549

**Published:** 2024-04-24

**Authors:** Rebecca Westley, Dawei Ma, Barbara J. Hawkins, C. Peter Constabel

**Affiliations:** Biology Department and Center for Forest Biology, University of Victoria, Victoria, BC, Canada

**Keywords:** *Populus*, proanthocyanidin, microelectrode ion flux measurement (MIFE), root cap, 4-dimethylaminocinnamaldehyde (DMACA), flavonoid

## Abstract

Condensed tannins are common in vegetative tissues of woody plants, including in roots. In hybrid poplar (*Populus tremula x alba;* also known as *P. x canescens*) CT assays indicated they were most concentrated in younger white roots and at the root tip. Furthermore, CT-specific staining of embedded tissue sections demonstrated accumulation in root cap cells and adjacent epidermal cells, as well as a more sporadic presence in cortex cells. In older, brown roots as well as roots with secondary growth (cork zone), CT concentration was significantly lower. The insoluble fraction of CTs was greatest in the cork zone. To determine if CT accumulation correlates with nutrient uptake in poplar roots, a microelectrode ion flux measurement (MIFE™) system was used to measure flux along the root axis. Greatest NH_4_^+^ uptake was measured near the root tip, but NO_3^-^_ and Ca^2+^ did not vary along the root length. In agreement with earlier work, providing poplars with ample nitrogen led to higher accumulation of CTs across root zones. To test the functional importance of CTs in roots directly, CT-modified transgenic plants could be important tools.

## 1 Introduction

Condensed tannins, (CTs, syn. proanthocyanidins) are the most widely distributed specialized plant metabolites in the plant kingdom. While in herbaceous plants they are typically restricted to the seed coat and other specialized tissues, CTs are highly prevalent and widespread in trees and woody plants and accumulate in all tissues, including leaves, bark and roots. CTs are typically high-molecular weight polyphenols synthesized from a branch of the well-known flavonoid pathway and are biosynthetically related to the anthocyanins. They consist of polymerized chains of 2-30 or more flavan-3-ol monomers, most commonly catechin and epicatechin, but also including gallocatechin and epigallocatechin (Barbehenn and Constabel, 2011). Like most other phenolic specialized metabolites, CTs are generally stored as soluble compounds in plant vacuoles. Nevertheless, depending on plant species and tissue, a significant fraction of CTs is not extractable and thus considered insoluble. It is assumed that CTs can become crosslinked to the cell wall or other structures, though mechanisms are unclear and exact structures are often undefined (Tarascou et al., 2010).

Condensed tannins can interact with diverse molecules via their numerous hydroxyl groups or via hydrophobic interactions of the phenolic rings (Quideau et al., 2011). This characteristic gives rise to the many potential molecular interactions of the CTs. Most commonly, they are known to bind proteins in solutions at neutral to low pH (Hagerman and Robbins, 1987; Barbehenn and Constabel, 2011). However, they can also bind to other polymers, including carbohydrate such as pectin, or chitin from fungal cell walls (Adamczyk et al., 2019). CTs also chelate positively charged ions including Fe^2+^ (Kimura and Wada, 1989; Elessawy et al., 2021) and Al^3+^ (Osawa et al., 2011; Schmidt et al., 2013). Furthermore, CTs have strong *in vitro* antioxidant capacity (Hagerman et al., 1998); by contrast under some conditions such as high pH, they can also act as pro-oxidants (Barbehenn and Constabel, 2011). Thus they have the potential for diverse biological effects (Constabel et al., 2014).

Leaf CTs are often believed to be general defenses against folivorous insect herbivores; however, for lepidopterans this is unlikely due to their high gut pH (Barbehenn and Constabel, 2011). Vertebrates and other animals with acidic guts can be negatively impacted by high concentrations of CTs, which bind and precipitate protein (Barbehenn and Constabel, 2011). CTs also have broad antimicrobial activity (Scalbert, 1991). *In planta*, this effect is demonstrated in experiments where high CT concentrations are associated with reduced disease intensity or infection (Ullah et al., 2017; Holeski et al., 2009). Other adaptive functions of CTs include their antioxidant capacity. We recently demonstrated their protective effects against oxidative stress in leaves caused by drought, UV-B, and herbicide exposure (Gourlay et al., 2022; Gourlay and Constabel, 2019).

The high CT content of woody plants also leads to CT accumulation in forest soils. Here they are known to modulate metabolic activities of soil microorganisms and alter the soil microbiome. This has direct impacts on nutrient cycling, and thus has important effects for forest carbon and nitrogen budgets (Hättenschwiler and Vitousek, 2000; Kraus et al., 2003; Coq et al., 2022). Recently, Adamczyk et al. (2019) demonstrated a role of tannins in stabilizing forest soil carbon by forming stable interactions with fungal necromass. Given the ecological dominance of forest ecosystems globally, the CTs are significant at the ecosystem scale (Whitham et al., 2006).

CTs are found in roots of many species of trees and woody plants (Endo et al., 2021), often at substantial concentration. For example, Dong et al. (2016) reported 6-16% CT content in a survey of roots of 15 Chinese temperate forest trees. Kraus et al. (2004) measured 1-3.2%DW in roots of ericaceous and coniferous shrubs and trees in the pygmy forest of coastal California. In *Populus tremuloides*, root CT concentration is reported between 1-5% DW depending on the study (Kosola et al., 2006; Stevens et al., 2014; Dettlaff et al., 2018). While the impacts of CTs when released into the soil have been well documented as outlined above, very little is known about functions of CTs within living roots and root systems. Similar to above-ground roles, CTs in roots may be important for defense against soil-borne pathogens and other microbes, or protect against herbivory. Furthermore, by virtue of their ability to chelate metal ions, in some species CTs are able to protect against toxicity from excess Al^3+^ (Osawa et al., 2011) or Fe^2+^ (Kimura and Wada, 1989) in the environment. It is also plausible that CTs can interact with nutrient cations, in particular NH_4_^+^, although this has not yet been investigated experimentally. Interestingly, low N availability has been observed to repress accumulation of CTs in a variety of species (Kraus et al., 2004; Stevens et al., 2014). These connections motivated us to investigate potential interactions of root CTs with nitrogen uptake.

Determining the distribution and localization of CTs in root tissues and cells is a critical first step for understanding potential functions of these compounds. Fine woody roots are typically composed of a white zone adjacent to the root tip, beyond which a brown zone as well as a cork zone can be distinguished (Peterson et al., 1999). The cork zone is formed by secondary growth, where the cortex is disrupted, and secondary xylem and a periderm are formed. The root tips and youngest regions of the root system are most active in water and mineral absorption (Hawkins et al., 2008), with older roots functioning in transport. Previous studies have localized CTs to various cell types of roots undergoing primary growth using 4-dimethylaminocinnamaldehyde (DMACA) staining (Kao et al., 2002; Hoffmann et al., 2012; Endo et al., 2021) and generally find CTs in root cap and epidermal cells near the root tips, with more sporadic localization in distal cortical cells.

Poplars, aspens, and cottonwoods (*Populus* spp., here collectively called poplars) are widely used for tree research, as they grow rapidly and can be clonally propagated. *Populus* has also become a molecular model system for tree molecular biology, genomics, and phenolic metabolism (Jansson and Douglas, 2007). Here, we systematically measured CT content, distribution and cellular localization along a developmental axis in hybrid *P. tremula x alba (syn. P. x canescens)* roots. In parallel, we measured NH_4_^+^, NO_3_^-^ and the cation Ca^2+^ net flux along the root axis using a microelectrode ion flux measurement (MIFE™) system, which allowed us to test for a correlation of CTs with N uptake.

## 2 Materials and methods

### 2.1 Plant material and growth conditions

*Populus tremula* x *alba* (clone INRA 717-1B4) plants were propagated from ~1 cm cuttings of *in vitro* plantlets and grown in half-strength Murashige-Skoog medium (Caisson Laboratories, UT, USA) supplemented with 0.5 μM indole-3-butyric acid (IBA). Plantlets were grown in culture in Magenta boxes for a minimum of eight weeks until they were approximately 8 cm in height. Plantlets were then transplanted into small seedling pots containing vermiculite, covered, and acclimated for three weeks in a mist-chamber. They were then repotted into vermiculite-filled, one-gallon round pots and moved into the greenhouse and grown for approximately 8 weeks. Plants used for histochemistry were fertilized three times per week with 100 mL of general purpose 20-20-20 Plant Prod^®^ NPK fertilizer (100 ppm nitrogen, phosphorus and potassium; Plant Products Co. Ltd, Brampton, ON, Canada), and watered with an equal volume of distilled H_2_O. Plants used for quantitative CT analysis and the nitrogen experiment were fertilized three times per week with a modified Long Ashton’s solution optimized for poplar growth (1 mM NH_4_NO_3_, 0.9 mM CaSO · 2H_2_O, 0.6 mM KH_2_PO_4_, 0.5 mM KCl, 0.04 mM K_2_PO_4_, 0.33 mM MgCl_2_ · 7 H_2_O, and 0.03 g L-1 standard micronutrient mix (Plant Products Co. Ltd, Brampton, CT ON, Canada), pH 5.6).

For N manipulation experiments, a *P. tremula x tremuloides* hybrid (clone INRA 353-38) was used. Plants propagated as above were grown in Sunshine Basic Mix #2 (Sungro, Seba Beach, AB, Canada) under three nitrogen fertilization treatments. Three plants were assigned to each nitrogen condition: 0.1 mM, 1 mM or 10 mM NH_4_NO_3_ considered to be low, medium (normal) and high nitrogen treatments, respectively. The plants were fertilized on alternate days with 100 mL of the desired NH_4_NO_3_ concentration within modified Long Ashton’s nutrient solution (0.9 mM CaCl_2_, 0.6 mM KH_2_PO_4_, 0.5 mM KCl, 0.04 mM K_2_HPO_4_, 0.3 mM MgSO_4_ x 7H_2_O and 0.03 g L^-1^ standard micronutrient mix (Plant Products Co. Ltd, Brampton, ON, Canada), pH 5.6). All other concentrations of nutrients were standardized in these solution. Plants were grown in greenhouse conditions for 12 weeks and watered with dH_2_O as necessary.

### 2.2 Quantification of condensed tannins

Roots were harvested after eight weeks of growth, divided into segments according to distance from root tip and root color, and then pooled into groups for each plant (n>30). Samples were frozen immediately in liquid nitrogen and then freeze-dried for three days. An aliquot of 8 mg was weighed from each pooled sample. For some samples (mainly root tips) where material was limited, the entire sample was weighed and extracted and the precise weight used in final calculations. Pooled root samples were extracted thrice in 100% MeOH by grinding freeze-dried samples in a PreCellys 24 homogenizer for two minutes using three 2 mm steel beads per sample, sonicating for ten minutes and centrifuging for five minutes at 1500 rpm. This procedure was followed using 1 x 1.5 mL, and 2 x 1 mL of 100% MeOH resulting in 3.5 mL of extract. The extract volume was made up to 5 mL to ensure it was within the linear range for soluble CT quantification. Soluble CTs were measured using the butanol-HCl assay (Porter et al., 1986) as described previously (Gourlay and Constabel, 2019). CTs purified from *P. tremula x tremuloides* leaf tissue (mean degree of polymerization ~9) was used as a standard, isolated and characterized as described by Preston and Trofymow (2015).

Insoluble CTs were quantified by depolymerizing CTs directly in the previously-extracted pellet. Six mL of 1-butanol:HCl (95:5) and 200 μL Fe reagent (Porter et al., 1986) were added directly to the dried pellet and heated to 95 oC for 40 minutes. 400 μL of MeOH was added to each sample before heating to correct for the volume of soluble extract used in the standard curve. Spectrophotometer readings were converted to CT concentration (μg mL-1) for the total sample using a standard curve prepared using purified Populus CTs (Mellway et al., 2009).

### 2.3 Histochemical analysis

Roots were carefully removed from plants, washed to remove potting soil, and cut into 1 cm sequential segments beginning at the root tip. Samples were immediately fixed at room temperature in modified Karnovsky’s fixative (25% glutaraldehyde and 16%) paraformaldehyde in 0.5 mM sodium phosphate buffer (PBS), pH 7.4). Samples were stored in fixative at 4 °C for up to four weeks before embedding. Samples were then rinsed three times for 30 minutes each with 0.5 mM PBS, pH 7.4, and dehydrated in sequential ethanol rinses (30%, 50%, 70%, 95%, and 100%) over the course of one day. Throughout the following week, plants were infiltrated with increasing concentrations of Technovit^®^ 7100 Glyco Methycrylate (Electron Microscopy Sciences) in 100% ethanol, before being cured.

Longitudinal sections (5 μm) were cut using a glass blade microtome (Sorval JB-4). Sections were dried onto slides and stained with 0.1% 4-dimethylaminocinnamaldehyde (DMACA) (w/v) in 0.5 M sulphuric acid in 1-butanol according to the method of Gutmann and Feucht (1991). The slides were covered in 1 mL of stain solution and heated on a Thermolyne Type 1000 hot plate (setting 2.5) for two minutes or until the stain began to evaporate and discolor. The slides were removed from the heat before bubbling occurred in the resin, rinsed three times for 30 s in 1- butanol or until all unbound stain was removed, and cover slips were then secured with PermountTM mounting medium. Sections were imaged within 24 hours on a transmitted light microscope (Zeiss [47-30-12-9902], Germany) fitted with a SPOT RTKE diagnostic 7.2 Color Mosaic camera. A deep burgundy stain indicated the presence of CTs, consistent with other reports of DMACA stained CTs in embedded samples hydrolyzed by hot sulphuric acid (Abeynayake et al., 2011; Gutmann, 1993).

### 2.4 Micro-electrode ion flux measurement analysis

Five clonal *P. tremula x alba* plants were grown for nutrient ion flux analysis. The Long Ashton’s solution was altered from previous experiments to match the measuring solution used during MIFE™ analysis, with overall concentrations of each ion similar to previous studies (Hawkins et al., 2014; 1 mM NH_4_NO_3_, 0.9 mM CaSO_4_ · 2H_2_O, 0.6 mM KH_2_PO_4_, 0.5 mM KCl, 0.04 mM K_2_PO_4_, 0.3 mM MgCl_2_ · 7 H_2_O and 0.03 g L^-1^ standard micronutrient mix, Plant Products Co. Ltd, Brampton, ON, Canada, pH 5.6). Plants were grown in pairs in a continuous rotation to ensure that they were all the same age when harvested after six weeks of growth. Healthy, individual roots were excised from the plant, and placed in aerated solution containing 500 μM NH_4_NO_3_ and 500 μM CaSO_4_ for 30 minutes prior to measurement to allow the roots to acclimate.

Borosilicate glass capillaries (1.5 mm diameter) were pulled, dried at 200 °C for five hours and silanized with tributylchlorosilane to make sterile, measuring electrodes. Once cooled, electrodes were backfilled with 200 mM NH_4_Cl for NH_4_^+^, 500 mM CaCl_2_ 2H_2_O for Ca^2+^, and 500 mM KNO_3_ 100 mM KCl for NO_3_^-^. Electrode tips were then filled with ion-selective resins: NH_4_^+^ and Ca^2+^ -selective cocktails (Fluka), and NO_3_^–^selective cocktail containing 0.5% methyltrididecylammoniumnitrate (MTDDA NO_3_^-^), 0.084% methyltriphenylphosphonium (MTPPB) and 99.4% n-phenyloctylether (NPOE) (Plassard et al., 2002). Electrodes were calibrated using a set of standards at pH 5.6. A reference electrode filled with 100 mM KCl in 1% agar and electrolyzed, chloride silver wire was placed in the solution for each measurement to complete the electrical current. Calibration was conducted at the beginning and end of each day to ensure readings were representative and reliable.

The electrodes were mounted in a specialized holder (MMT-5, Narishige, Tokyo, Japan) providing three-dimensional positioning. A maximum distance of 20 μm was recorded between the electrodes and the root surface, and 4 μm between the electrode tips. Roots were secured and placed in the measuring chamber with fresh measuring solution (500 μM NH_4_NO_3_ and 500 μM CaSO_4_). The chamber was moved by a computer-controlled manipulator (PatchMan NP2, Eppendorf AG, Hamburg, Germany). During flux measurements, the MIFE™ computer caused the chamber to gently oscillate over a distance of 40 μm, back and forth from the root surface in a 10 s square-wave cycle. The concentration of each ion was calculated from its electrochemical potential at the two positions and ion net flux calculated from the difference in concentration between the two positions (Shabala et al., 1997).

Flux measurements were taken at four discrete positions along the root in the white zone: at the root tip (<2 mm), and three positions within the maturation zone of the white root zone (0.5 cm, 1.5 cm and 3.5 cm from the root tip). These root distances were chosen as they included the zones where uptake is expected to be highest (Hawkins et al., 2008; 2014).

### 2.5 Statistical analysis

Data was analyzed and graphed using R v.3.0.3. Once model assumptions and normality were checked, data was statistically analyzed using a two-sample T-test or one-way analysis of variance (ANOVA) with Tukey’s Honestly Significant Difference (HSD) test.

## 3 Results

### 3.1 Condensed tannin content is greatest at the root tip and within the white zone of roots

**Figure 1:**
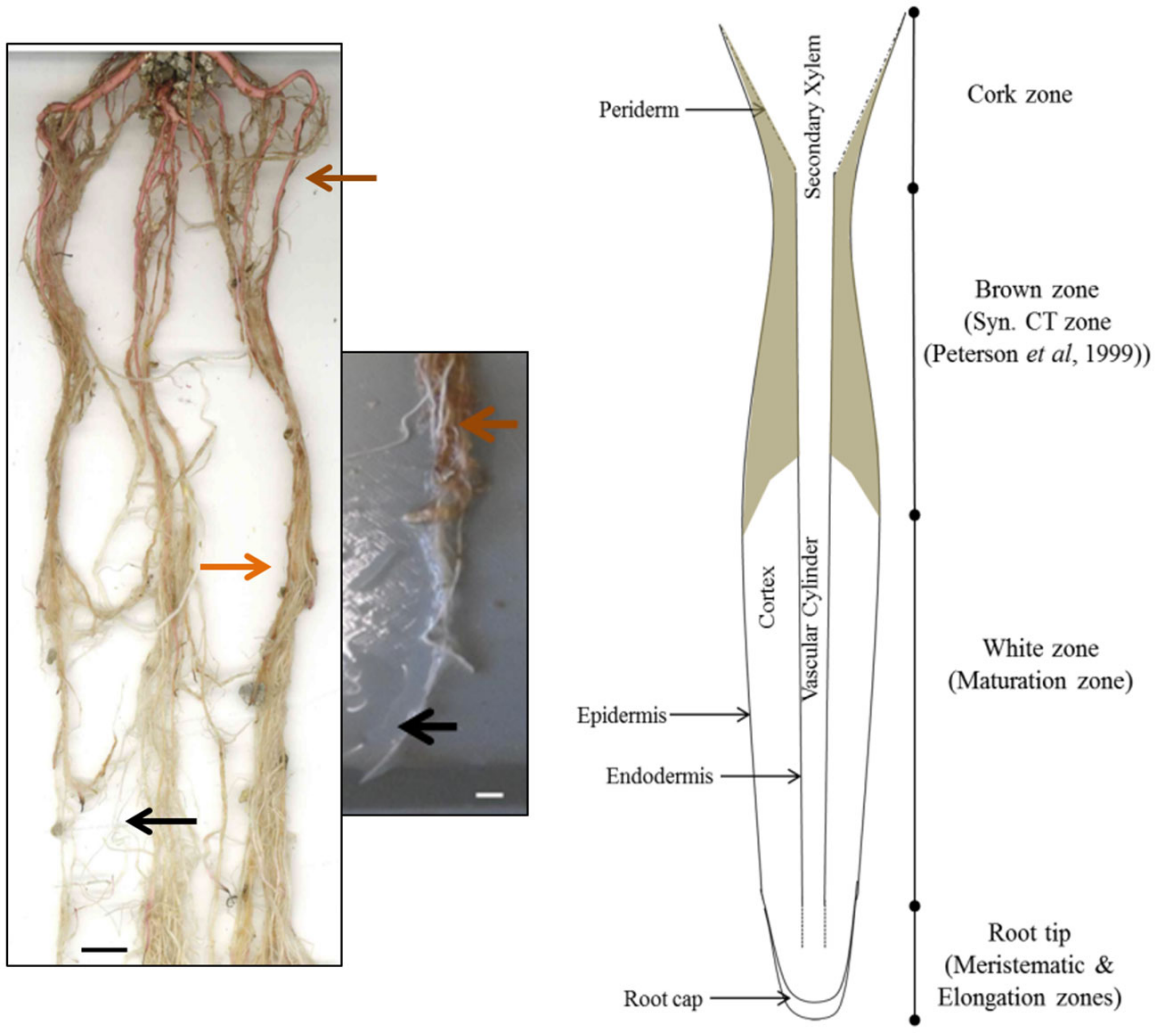
Images and cartoon of typical *Populus tremula* x *alba* roots after eight weeks of growth showing white zone and transition to brown zone. Black arrows indicates typical white zone; orange arrows show brown zone of roots prior to secondary growth. Brown arrow indicates the cork zone. Scale bar = 1 cm.

In order to determine the overall distribution of CTs in roots, root systems from greenhouse-grown poplar were carefully removed from pots and soil and root zones separated and dissected. Root systems were white at the actively growing regions, with older sections becoming brown, and developing obvious secondary growth towards the crown (**Figure 1**). We used both color and root diameter to divide root systems into four zones similar to the classification of Peterson et al. (1999): root tips (meristematic and elongation zone; <0.5 cm), white zone (maturation zone; 1.0-12 cm), brown zone (without periderm; 13-20 cm), and cork zone (with periderm; >20 cm). Any root tips that showed necrosis were excluded.

**Figure 2:**
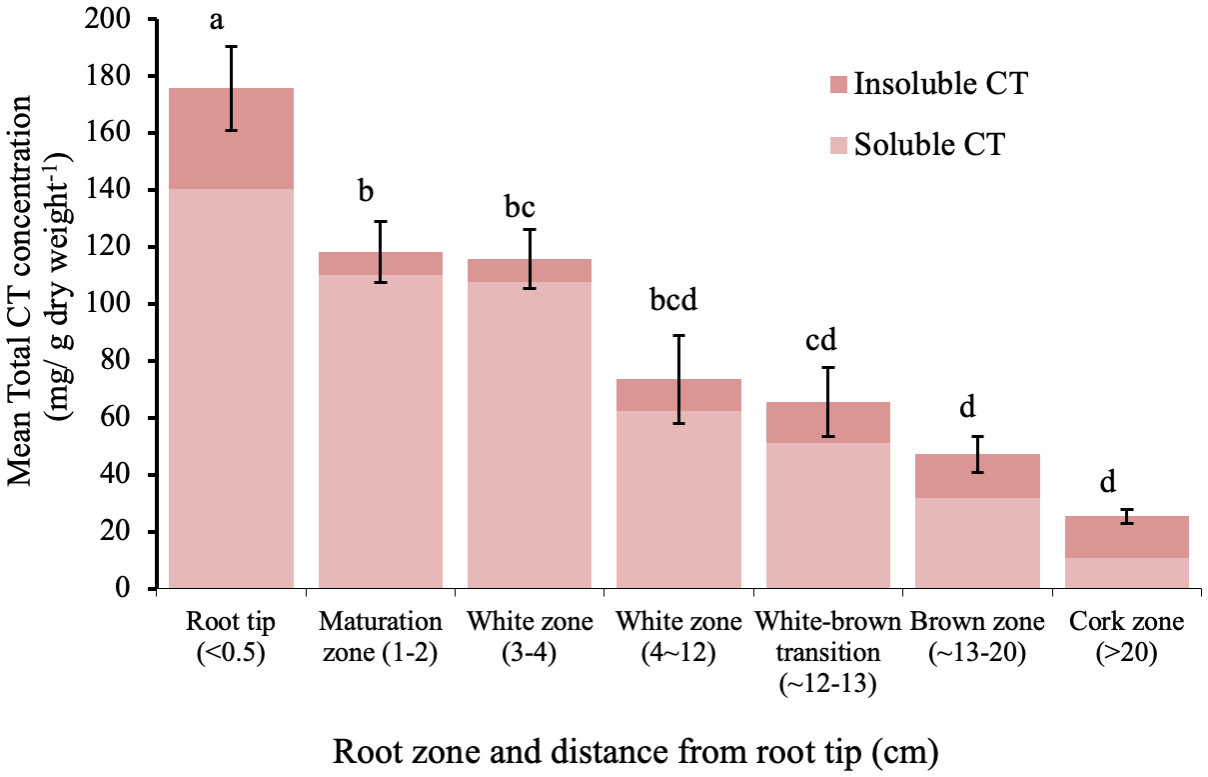
Mean soluble and insoluble CT concentrations in zones along the root axis in *P. tremula* x *alba*. Root zone samples were pooled individually by plant for each of five plants. Different letters indicated significant differences (Tukey HSD p<0.05). Error bars indicate SE (n=5) for total CT concentration.

The sum of soluble and insoluble CT concentration was highest in the root tips and within the white zone, and then decreased with increasing distance from the root tip (**Figure 2**). In the youngest parts of the roots (< 5 cm from the root tip), CTs accumulated to more than 100 mg/g DW; this is as much as 20-fold greater than the CT concentration in leaves of greenhouse grown plants. Older parts of the root system contained approximately 25-50 mg/g DW. The lowest CT content was found in the oldest zone, which showed secondary growth - the cork zone. Soluble CTs comprised the predominant fraction of CTs in all regions except in the cork zone, where insoluble CTs were predominant (**Figure 2**).

### 3.2 Histochemical analysis indicates a distinct pattern of CT accumulation in epidermal and subepidermal tissues

**Figure 3:**
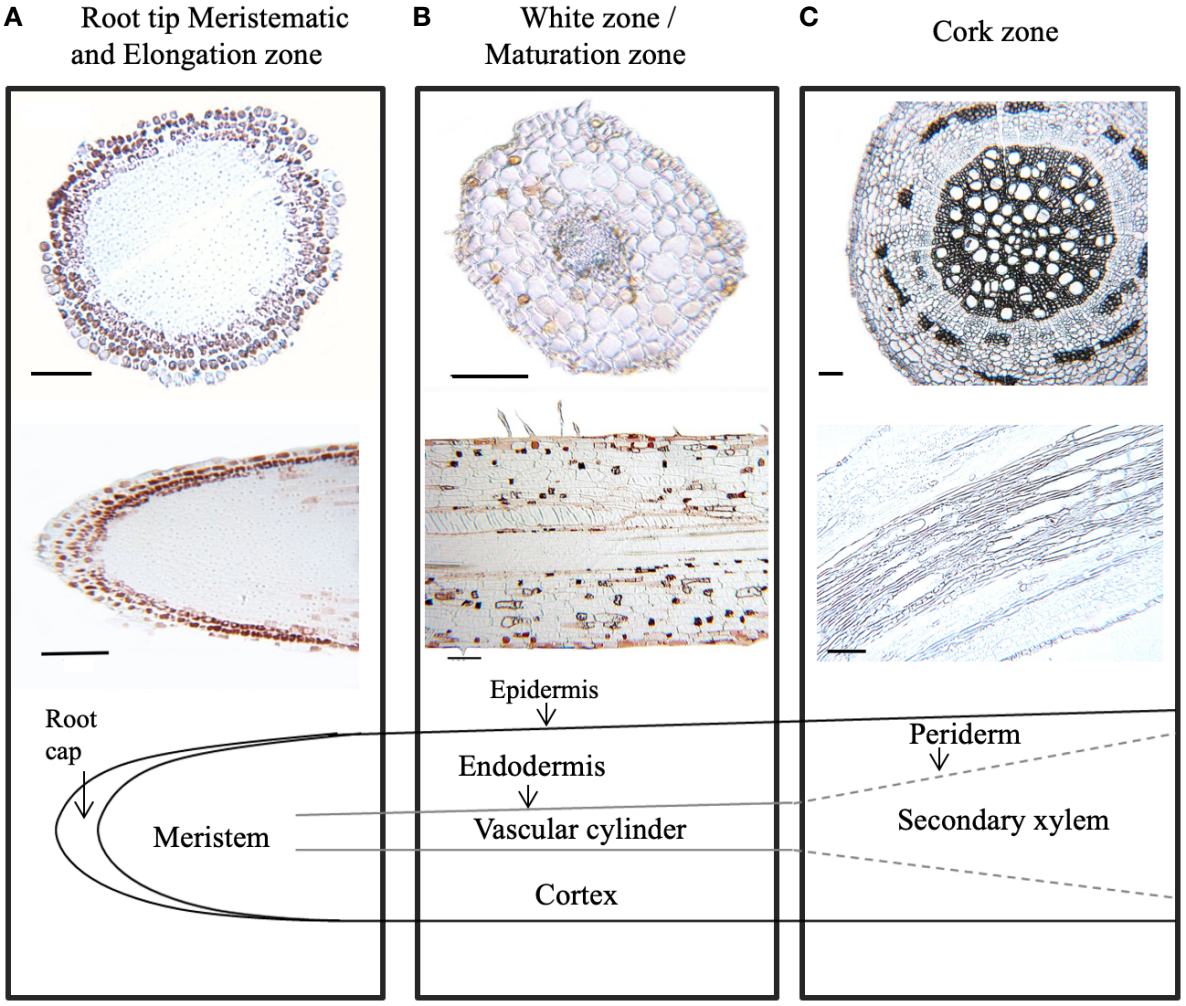
Localization of condensed tannins in *P. tremula x alba* roots visualized by DMACA staining of longitudinal and cross root sections. CTs stain with reddish-brown color under these conditions. **(A)** Root tip (< 2 mm from root). **(B)** White zone/maturation zone (1cm back from root tip). **(C)** cork zone (> 20 cm back from root tip). Scale bar = 100µm.

To further define areas of CT accumulation at the cellular level, embedded tissues from excised root samples from different root zones were stained with 4-dimethylaminocinnamaldehyde (DMACA). In embedded tissue sections, this stain colors CTs reddish brown rather than blue (Abeynayake et al., 2011). In our root sections, CTs were strongly stained in several cell layers of the root cap, but not in the underlying smaller meristematic cells (**Figure 3A**). In the white zone immediately adjacent (~ 1 cm from tip), CTs were detected in the epidermal cell layers just behind the root cap. Staining was also seen sporadically in cortical cells (**Figure 3B**). Some DMACA positive staining was observed in the endodermis, but not within vascular tissue of the stele. In older roots, where secondary xylem expands laterally and begins to disrupt the cortex, only very low levels of staining were observed (**Figure 3C**). Overall, the pattern of DMACA staining was consistent with the chemical analysis: the most intense staining of CTs was seen in the tips and younger sections of the roots, predominantly in epidermal and subepidermal regions around the tip.

**Figure 4:**
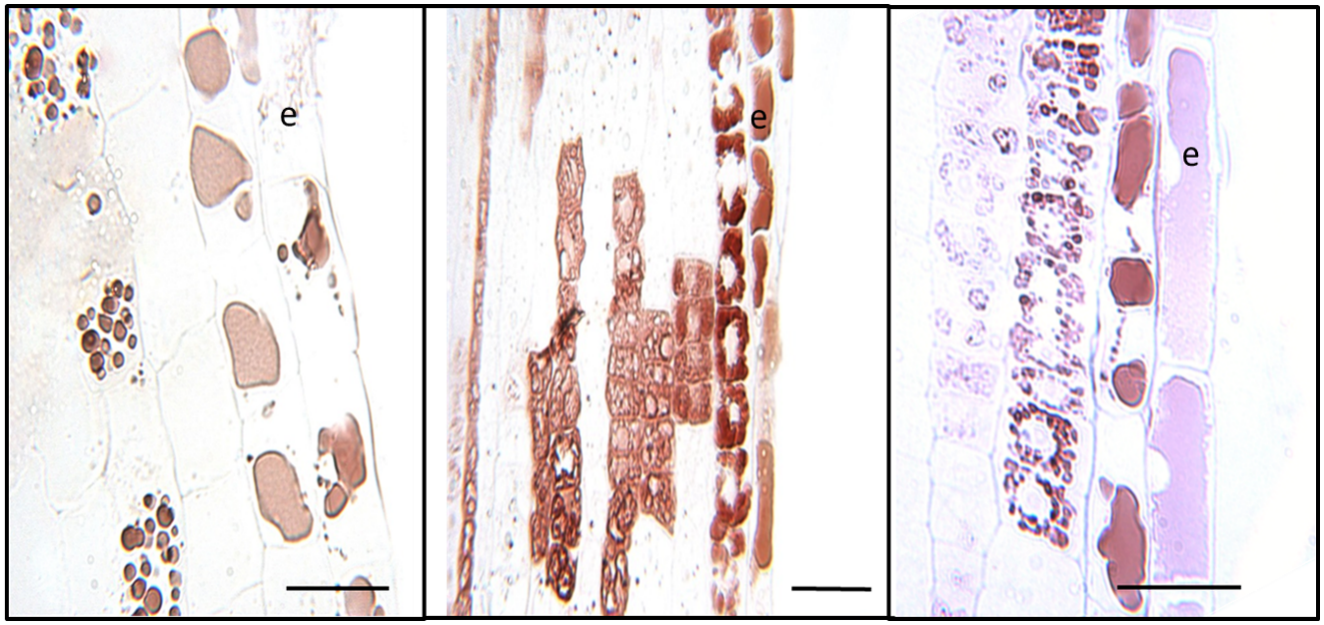
Cellular localization of condensed tannins of *P. tremula x alba.* Longitudinal sections of roots 5-10mm from root tip in the white zone were embedded, sectioned and stained with DMACA as described under Materials and Methods. CTs stain with reddish-brown color under these conditions. Epidermal cells are indicated by ‘e’. Scale bars=25µm.

At higher magnification, distinct patterns of CT accumulation distribution were visible within different cell types within the white zone. In the outer epidermal layer of the young white root, CT staining was typically observed in large vacuoles filling most of the intracellular space (**Figure 4**). By contrast, in cortical cells below the epidermal layer, CTs tended to occur in smaller, more concentrated vacuole-like structures. In many cases, these CT-containing structures were arranged around the periphery of the cells, possibly around a central zone or structure, or were found throughout the cell. CTs were not present in all cortical cells but occurred somewhat sporadically, as we had noted at lower magnification (**Figure 3**). This pattern was observed consistently in multiple sections observed within this region.

### 3.3 Interactions of N uptake and nutrition with CT accumulation in different root zones

**Figure 5:**
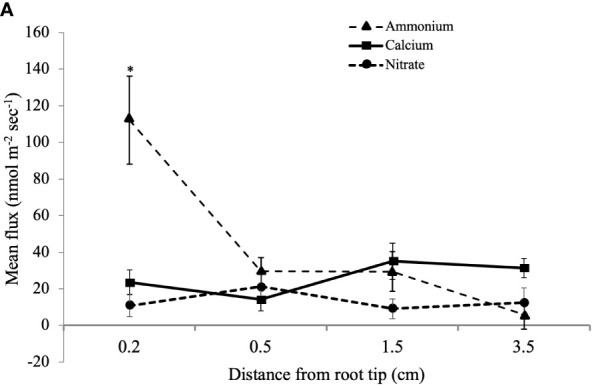
Mean influx of NH_4_^+^, Ca^2+^, and NO_3_^-^ ions into white zone roots of *P. tremula x alba* at defined distances from the root tip. Mean ion fluxes (+/- SE) were measured in young poplar roots at a defined distance from root tip using a MIFE system as described in Materials and Methods. *indicates significantly different means from other measurements for the same ion (p<0.001).

MIFE™ technology is a non-invasive method to measure net fluxes of specific nutrient ions at precise root locations (Shabala and Newman, 1997; Shabala et al., 1997). To determine if there is a potential interaction of CTs with N uptake, we looked for correlations of N fluxes with CT accumulation. We measured the net flux of NO_3_- and NH_4_+ along the longitudinal axis of young poplar roots using a MIFE system. Since the root tip and youngest part of the root is most active in nutrient acquisition (Hawkins et al., 2014), we focused on the white zone and root tip. Ion net fluxes were measured for eight minutes at each of four positions along the root axis to ensure that a reliable average flux measurement was gained for each replicate. Five plants were grown and three roots were measured per plant. The highest NH_4_^+^ net fluxes were observed at the root tip, which were significantly greater than fluxes at more distal positions (**Figure 5**). NO_3_- uptake was lower than NH_4_^+^ flux at the root tip and did not vary significantly along the length of the root. We also measured the net fluxes of another cation, Ca^2+^, at the three positions, and these also did not vary with distance from the root tip.

**Figure 6:**
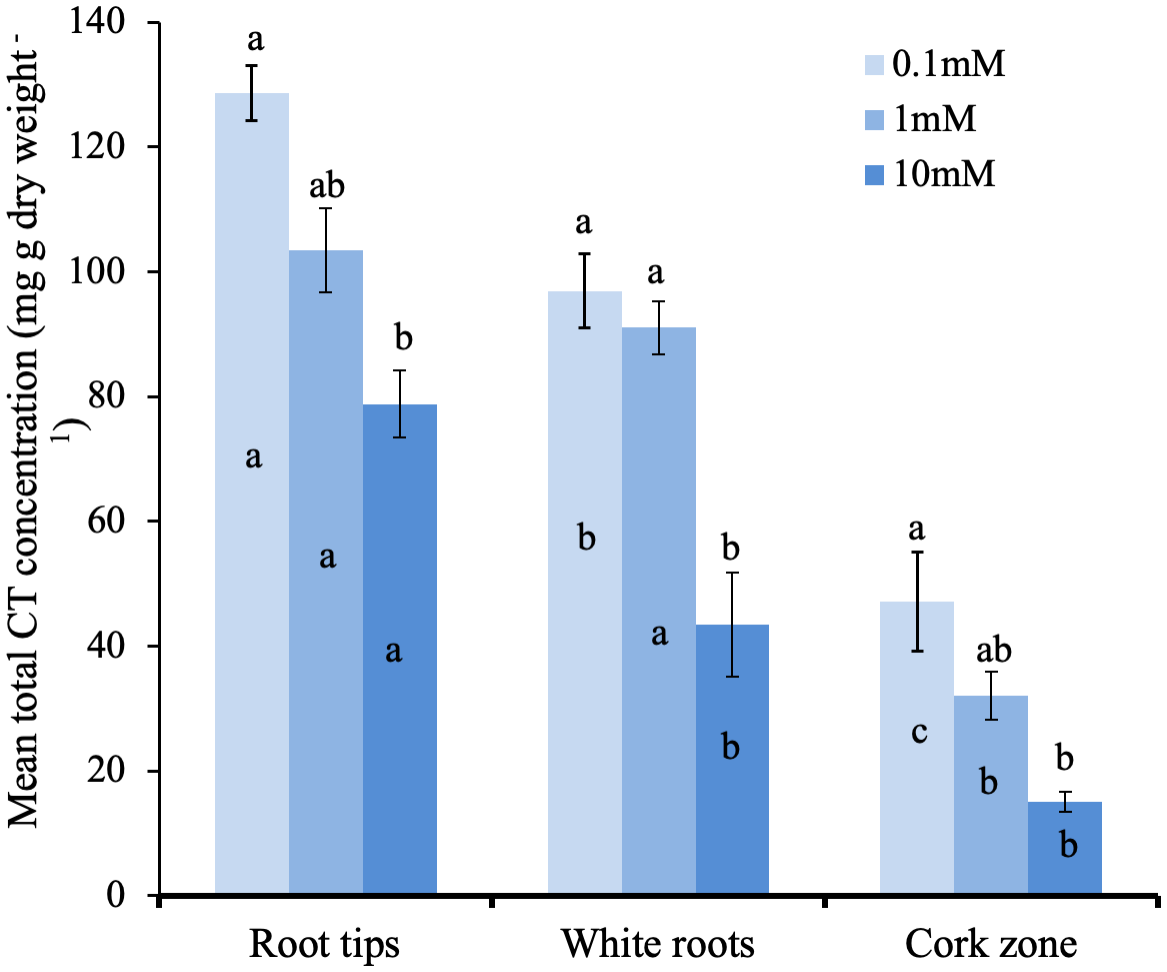
Total CT concentrations in distinct growth zones of *P. tremula x P. tremuloides* roots grown with 0.1, 1.0, or 10 mM N. Small letters indicate significantly different means (Tukey HSD, P<0.05). Bars show means ± SE (n=3). Letters above bars are Tukey pairwise comparisons for differing nitrogen treatments within each root zone. Letters within bars are pairwise comparison between different root sections with the same nitrogen treatment.

We next tested if CT concentration in the different root zones is influenced by external N supply. Young potted poplar plants were fertilized with a modified Long Ashton nutrient solution that provided 0.1, 1.0 or 10 mM NH_4_NO_3_ (low, medium, and high N). After the six-week experimental period, the impact of N limitation at the lowest N level was shown by reduced plant growth and a lighter leaf color compared to plants grown at medium and high N availability. Roots were excised and samples taken from the tip, white, and brown zones for CT assays. Under all three N treatments, we observed the same pattern: highest CT concentration at the root tip, which decreased with increasing distance from the tip in the white and brown zones (**Figure 6**). Within each root zone, the highest CT concentration was measured in roots grown with 0.1mM NH_4_NO_3_; roots of plants grown under intermediate N levels showed intermediate CT concentration, and the lowest CT concentration was measured for all three root zones in plants supplied with 10 mM NH_4_NO_3_. In the white zone, CT concentration was two-fold lower at the highest compared to lowest N availability. This confirmed the repressive effect of N on CT metabolism, and demonstrated this effect across all three root zones.

## 4 Discussion

The importance of CTs as flexible adaptations of woody plants to biotic and abiotic stresses has emerged in recent years (Constabel et al., 2014; Gourlay et al., 2022), but their functions in roots are not well understood. As a first step in a functional analysis of root CTs, we studied CT localization and distribution in the poplar hybrid *P. tremula* x *alba*. Based on both CT assays and DMACA staining, our work demonstrates the greatest concentration of CTs in the root tip and proximal regions of the white zone, decreasing with increasing distance from the apex. This gradient of decreasing CTs along the root axis was not influenced by altered N-availability, although overall CT concentrations were reduced with the highest N treatment. In embedded sections, CT-specific staining showed a distinct localization of CTs to cells of the root cap and immediately adjacent epidermal cells, with some sporadic accumulation in cortical cells. Using a microelectrode ion flux measurement (MIFE™) system, we also found the root tip to be the most active zone for NH_4_+ uptake; by contrast, NO3- and Ca^2+^ uptake did not vary significantly along the root axis. To our knowledge, this is the first report testing for a correlation of CT localization in roots with nutrient uptake.

The localization of CTs in the root apical region in the white root zone observed here is consistent with reports from other species. Early work with woody species in the *Rosaceae* also suggested that root tips have particularly high CT content, with accumulation in the root cap and epidermal layers of the elongation zone (Weiss et al., 1997; Hoffmann et al., 2012). Likewise, in *Populus tremuloides* roots, Kao et al. (2002) previously reported CTs in the root cap and epidermal layers near the root tip, as well as in randomly distributed cortical cells closer to the apex and cell division zone. By contrast, in other species a CT distribution in older parts of the root was reported. For example, in *Pinus* and *Eucalyptus* roots, CTs were reported primarily from older, brown regions of the root system (McKenzie and Peterson, 1995). The authors named this the ‘tannin zone’; however, this is not consistent with our findings and others that report the highest CT levels in the tip and adjacent regions. Endo et al. (2021) analyzed root cross-sections from primary and higher order roots from 20 different temperate woody plants, and found a diverse distribution of CTs. Most species studied stained for DMACA most consistently in the first order (youngest) roots in different cell types including epidermal, phloem and cortex cells. Since mostly older roots were used in that work, it is difficult to compare directly without taking into consideration developmental stages or tissue age; importantly, our work does demonstrate the breadth of cell types and diversity of CT accumulation patterns in different species. Endo et al. (2021) also concluded that trees with ectomycorrhizal associations tended to have higher CT content than those with arbuscular mycorrhizal associations.

The intracellular patterns of CT accumulation as visualized by DMACA at higher magnification is intriguing. In the epidermal cells near the root tip, stain is observed throughout the large central vacuole, whereas in other cells we see mini-vacuoles. The vacuolar accumulation of CTs is broadly known, and has been observed directly using osmium-stained transmission electron microscopy (Lees et al., 1993; 1995). These studies investigated CT localization in apple seeds and peel, as well as leaves and other tissues in sainfoin, a forage crop. All show a diversity of vacuolar accumulation patterns that includes reticulated, net-like patterns, round globules, or a band of CTs that accumulates around the tonoplast. While these studies do not report on CTs in roots, the diversity of CT accumulation patterns in different cells is intriguing and consistent with CT accumulation patterns we detected here. How these accumulation patterns relate, and if they represent different stages in CT synthesis and accumulation, are interesting questions that merit further study.

It should be noted that recently developed fluorescence-based DMACA localization methods in poplar roots provide direct evidence of CT accumulation in cell walls (CW), as well as within the cells (Chowdhury et al., 2023). The CW signal likely represents the unextractable CT fraction often observed in plant tissues (Tarascou et al., 2010). Our CT assays confirm that a substantial proportion of CTs are insoluble. Interestingly, Chowdhury et al. (2023) also show a tissue-age related accumulation of CT. Likewise, our chemical data demonstrate a greater proportion of insoluble vs soluble CTs in the older, cork zone roots. This could be the result of gradual cross-linking with cell walls during root development. Taken together, this pattern suggests that CT accumulation are more dynamic than previously thought.

The negative effect of N supply on CT accumulation we observed in roots is consistent with the pattern observed in other species, including in poplar (Stevens et al., 2014). Our observation of this negative association in all three root zones further underlines that this reflects a fundamental metabolic adaptation. However, to date no consistent physiological function or adaptive function of CT induction under low N supply (or repression of CT synthesis under high N) has emerged. The carbon-nutrient balance hypothesis proposes that CTs act as an internal carbon sink under nutrient limiting conditions and has been much discussed, but has not received broad experimental support (Bryant et al., 1983; Stamp, 2003). Alternative explanations invoke the antioxidant potential of CTs. For example, in leaves low N availability may lead to a reduced capacity for photosynthetic electron transport, and thus lead to a greater production of reactive oxygen species. Together with the ability of CTs to protect against oxidative stresses in leaves (Gourlay et al., 2022), this could explain the stimulation of leaf CT synthesis under N stress. How this could apply to below-ground effects is not clear, but oxidative stress can also be caused by heavy metals in soil. We speculate that ample N supply can facilitate enzymatic and protein-based adaptations to below ground oxidative stress. In the absence of N, carbon-rich phenolics such as tannins may provide alternative anti-oxidant mechanisms.

Although preliminary, the correlation of CT content and NH_4_+ uptake could be suggestive of a role of CTs in modulating some cation movement into the root. However, additional work is needed to better understand how these patterns may vary seasonally or under other environmental conditions. Plants with modified root CT content (Liu et al., 2023) would be useful tools in testing this as well as other potential functions of CTs in roots. Since mycorrhizae are critical components of nutrient availability for many forest trees, future work will include studies of the roles of CTs in regulating these ecological interactions.

## Data availability statement

The original contributions presented in the study are included in the article/supplementary materials, further inquiries can be directed to the corresponding author/s.

## Author contributions

RW: Conceptualization, Methodology, Visualization, Writing – original draft, Writing – review & editing, Formal analysis, Investigation. DM: Investigation, Methodology, Resources, Writing – review & editing. BJH: Methodology, Writing – review & editing, Funding acquisition. CPC: Conceptualization, Funding acquisition, Methodology, Resources, Supervision, Visualization, Writing – original draft, Writing – review & editing.
